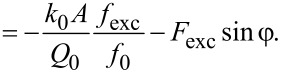# Quantitative dynamic force microscopy with inclined tip oscillation

**DOI:** 10.3762/bjnano.13.53

**Published:** 2022-07-06

**Authors:** Philipp Rahe, Daniel Heile, Reinhard Olbrich, Michael Reichling

**Affiliations:** 1 Fachbereich Physik, Universität Osnabrück, Barbarastrasse 7, 49076 Osnabrück, Germany

**Keywords:** atomic force microscopy, cantilever, quantitative force measurement, sampling path

## Abstract

In the mathematical description of dynamic atomic force microscopy (AFM), the relation between the tip–surface normal interaction force, the measurement observables, and the probe excitation parameters is defined by an average of the normal force along the sampling path over the oscillation cycle. Usually, it is tacitly assumed that tip oscillation and force data recording follows the same path perpendicular to the surface. Experimentally, however, the sampling path representing the tip oscillating trajectory is often inclined with respect to the surface normal and the data recording path. Here, we extend the mathematical description of dynamic AFM to include the case of an inclined sampling path. We find that the inclination of the tip movement can have critical consequences for data interpretation, especially for measurements on nanostructured surfaces exhibiting significant lateral force components. Inclination effects are illustrated by simulation results that resemble the representative experimental conditions of measuring a heterogeneous atomic surface. We propose to measure the AFM observables along a path parallel to the oscillation direction in order to reliably recover the force along this direction.

## Introduction

Atomic force microscopy (AFM) is a quantitative technique that allows for probing the force field above a surface in one, two, or three dimensions. While imaging in a plane parallel to the surface provides nanoscale and atomic structural information [1], force curves, usually acquired along a recording path perpendicular to the surface, provide quantitative information about the details of the tip–surface interaction when properly analysed [2]. Recently, a universal description of quantitative dynamic force microscopy based on the harmonic approximation has been developed [3], yielding three central equations that link the physical interaction parameters force 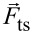 and damping 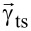 with the measurement observables static deflection *q_s_*, oscillation amplitude *A*, and phase φ as well as the excitation parameters frequency *f*_exc_ and force *F*_exc_. This theory specifically predicts the distant-dependent frequency shift of a tip moved perpendicular to a surface for a given force curve. Inversion formulae are available that allow for the extraction of the interaction force from measured frequency-shift data [4–5].

A tacit assumption of all prevalent algorithms for force inversion is that the axis of data acquisition (herein denoted as the recording path, usually the axis of the piezo scanner, *z*_p_) is parallel to the axis of the oscillation (herein denoted as the sampling path). However, in a typical experimental setup this is not the case. Instead, angles of 10° to 20° between these two directions are often present for technical reasons. Consequences of this inclined AFM cantilever mount have been identified before, in particular for atomic force microscopy performed in static (“contact”) mode where an effective spring constant [6–8] has been introduced and a torque [9–10] as well as load [11] correction has been applied. Additionally, a tilted cantilever has been found to lead to a modification of the tip–sample convolution [12], to enhance the sensitivity of the measurement to the probe side [13], and to influence results of multifrequency AFM and Kelvin probe force microscopy [14]. In the presence of a viscous damping layer, in-plane dissipation mechanisms have been found to cause systematic changes of the phase shift in amplitude-modulation AFM depending on the cantilever inclination [15]. Furthermore, it has been proposed to use the presence of a lateral component in the tip oscillation path for the investigation of in-plane material properties, such as the in-plane shear modulus [16]. Last, the influence of the inclination between oscillation direction and surface plane has been used in lateral force microscopy to determine the probe oscillation amplitude [17].

Here, we extend the established mathematical description for dynamic atomic force microscopy [3] by including free orientations of the tip sampling and data recording paths. The resulting formulae are discussed and implications for precise force measurements [2] are identified and quantified. Most importantly, the data acquisition with an inclined tip sampling path requires modifications of the experimental procedures and data analysis protocols for force measurements to avoid systematic errors in the interpretation of force curve and imaging data.

## Results and Discussion

### Sensor positioning, sensor displacement, and tip position

Prerequisite to quantitative force microscopy is a precise definition of the involved probe and sample coordinates as well as probe dynamical parameters that are outlined in the following.

**Figure 1:**
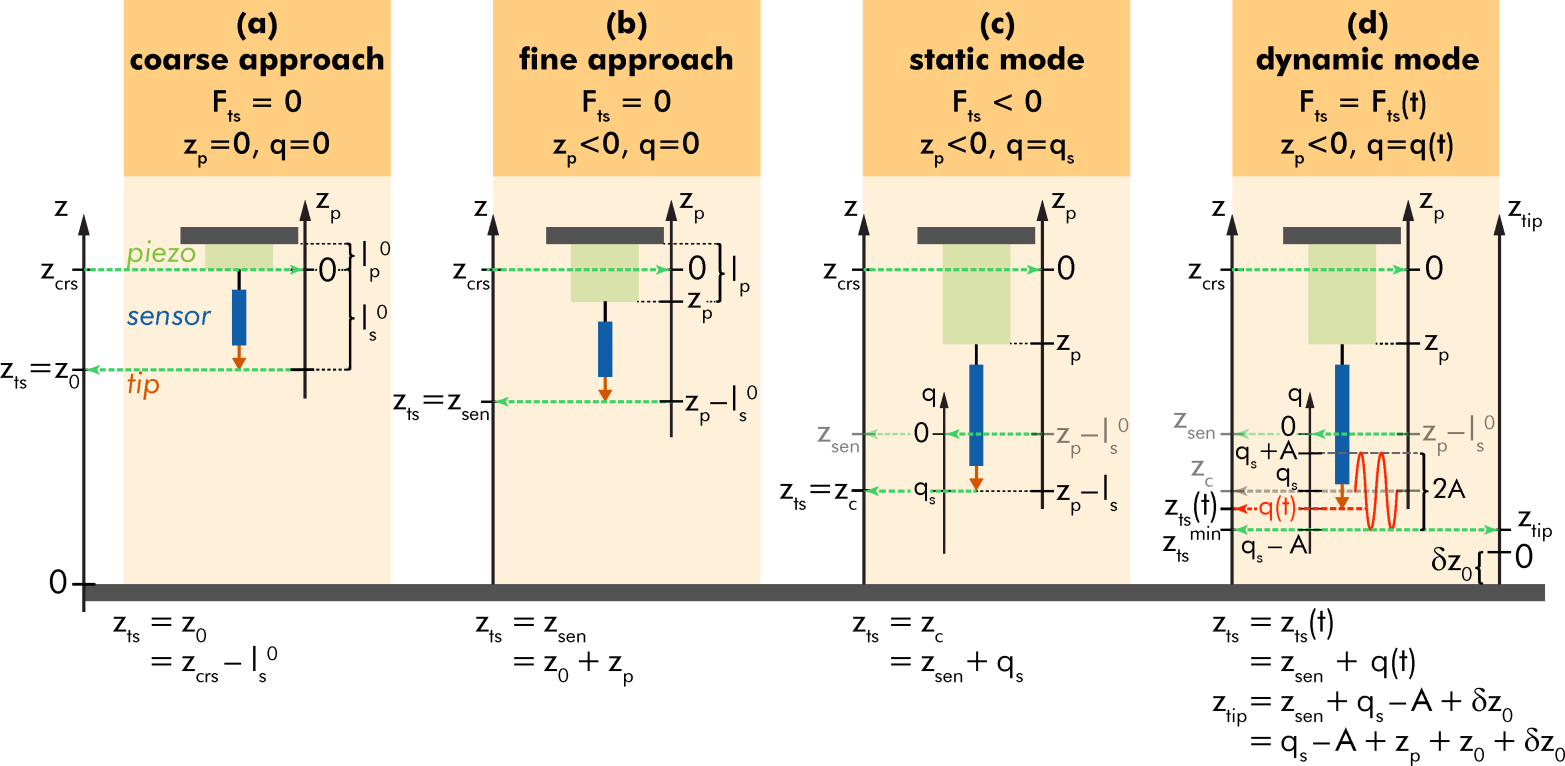
Coordinates describing the one-dimensional tip positioning and movement. See main text for description.

In dynamic AFM, the force 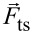 acting between a sharp tip and the surface under investigation is measured as a function of the tip position 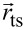 that is usually described in Cartesian coordinates with the origin placed in the sample surface and the *z*-axis with unit vector 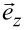 oriented perpendicular to the surface as shown in Figure 1. Lateral movements of the tip as applied for imaging are associated with the *x* and *y* axes, while the tip–surface distance *z*_ts_ is measured along the *z*-axis. In most AFM implementations, the force measurement is restricted to nominally measuring the normal component of the tip–sample force 
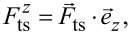
 often denoted by *F_N_*. The ideal force curve is a measurement of 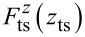 while the measurement of 
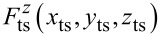
 is referred to as force mapping.

To measure the tip–surface force in a dynamic measurement, the force probe acts as a high-Q oscillator and elastically responds to 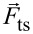 by static and dynamic displacement described by 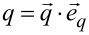 with 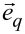 being the unit vector along the tip sampling path. This path is usually straight and assumed to be strictly parallel to 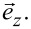 Furthermore, we assume an infinitely stiff sensor in directions perpendicular to 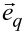 as well as a linear sensor response along 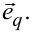 Then, the static probe response follows Hooke’s law 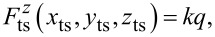 with *k* being the static sensor force constant [18]. In dynamic mode operation, the sensor is excited to periodic displacement *q*(*t*) = *q*(*t* + 1/*f*_exc_) along the *q*-axis at an excitation frequency *f*_exc_.

To bring the tip in the desired range of interaction with the surface and to perform the movements required for imaging, force mapping, and taking force curves, the sensor is moved by coarse and fine positioning elements acting at least along the *z*-axis. To accomplish this, the sensor is attached to a piezo element allowing for fine positioning that, in turn, is attached to a coarse positioning system. The respective sensor positioning movements, the sensor oscillation, and its response to the mean tip–surface force are illustrated in the sketches of Figure 1 for the case of parallel tip sampling and data recording paths.

Initially, the sensor assembly is moved towards the surface by the coarse positioning system so that the relaxed piezo rests at position *z*_crs_ and the tip at its starting position *z*_0_ (Figure 1a). In its relaxed state, the *z* piezo and the force sensor have a length of 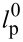 and 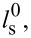 respectively. Applying a voltage to the *z*-piezo results in an extension of the piezo length *l*_p_ that is described as a piezo position *z*_p_ on the separate axis *z*_p_ with unit vector 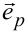 and with the origin chosen to coincide with the *z*_crs_ position (Figure 1b). As the unit vectors 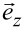 and 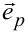 are chosen to point into the same direction, a piezo extension *z*_p_ *<* 0 results in an approach of the tip towards the surface while *z*_p_ *>* 0 indicates a tip retraction. Coarse and fine approach define the sensor position *z*_sen_ = *z*_0_ + *z*_p_, which is at this point identical to the tip position (tip–sample distance) *z*_ts_ as the force *F*_ts_ acting on the tip is unmeasurably small for sufficiently large *z*_ts_. Upon further approach of the sensor, however, the tip experiences a measureable force, yielding a static sensor displacement *q_s_* described on the *q*-axis with the origin chosen at *z*_sen_, corresponding to the tip centre position *z*_c_ = *z*_sen_ + *q_s_* (Figure 1c). As 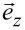 and 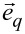 point in the same direction, a sensor displacement *q <* 0 corresponds to a tip movement towards the surface. Note that the tip centre position *z*_c_ cannot easily be set or determined as the static sensor displacement is governed by the a priori unknown force curve. Furthermore, *q_s_* is usually so small that it is at or beyond the limit of detectability for most NC-AFM implementations. In dynamic NC-AFM operation, the sensor oscillates with an amplitude *A* symmetrical to the static displacement *q_s_* with turning points *q_s_* + *A* and *q_s_* − *A* (Figure 1d). The momentary tip position at time *t* can either be described as the displacement *q*(*t*) or as the position *z*_ts_(*t*), whereby the lower turning point 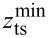 is the point of strongest tip–surface interaction.

While the tip position and sensor dynamics can principally be well described by the respective positions on the *z*-axis, this axis is practically of limited use as its zero point cannot be defined or determined in a reasonable way. This is due to the fact that neither *z*_crs_ nor 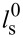 can be determined with atomic-scale precision, which would be needed for properly taking into account the force curve 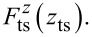 Furthermore, it is conceptually difficult to define the position of the surface at the atomic scale. As every force curve acquired on a surface diverges for *z*_ts_ → 0, the natural choice of the *z*-axis origin would be the *z* value approached by the diverging force. This point is, however, experimentally not accessible. Instead, precise values for the piezo position *z*_p_ and the sensor displacement *q*(*t*) are experimentally available. To derive a force–distance curve experimentally, the usual procedure is therefore to apply dynamic AFM and to measure the distance-dependent shift in frequency, Δ*f*(*z*_p_), of the sensor excitation frequency *f*_exc_ that results when phase resonance for the sensor oscillation is maintained throughout the measurement [19]. The resulting curve Δ*f*(*z*_p_) is a convolution of the covered part of the force curve 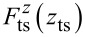 and a kernel depending on the stabilised sensor oscillation amplitude *A*. A sophisticated analysis of the Δ*f*(*z*_p_) curves measured with different oscillation amplitudes *A* yields a precise result [2] for the force curve, yet with an arbitrary origin along the *z*-axis. In theoretical modelling and analysis of tip–sample interactions, it has been established as a standard to represent force curves as 
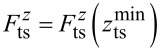
 [4–5]. As 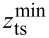 is practically not accessible, for the representation of force curves we introduce an axis *z*_tip_ that is identical to the *z*-axis except for an unknown offset δ*z*_0_ for the tip starting position and describe a force curve resulting from the analysis of measured data as 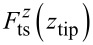 where 
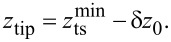


### Geometry for the inclined sampling path

**Figure 2:**
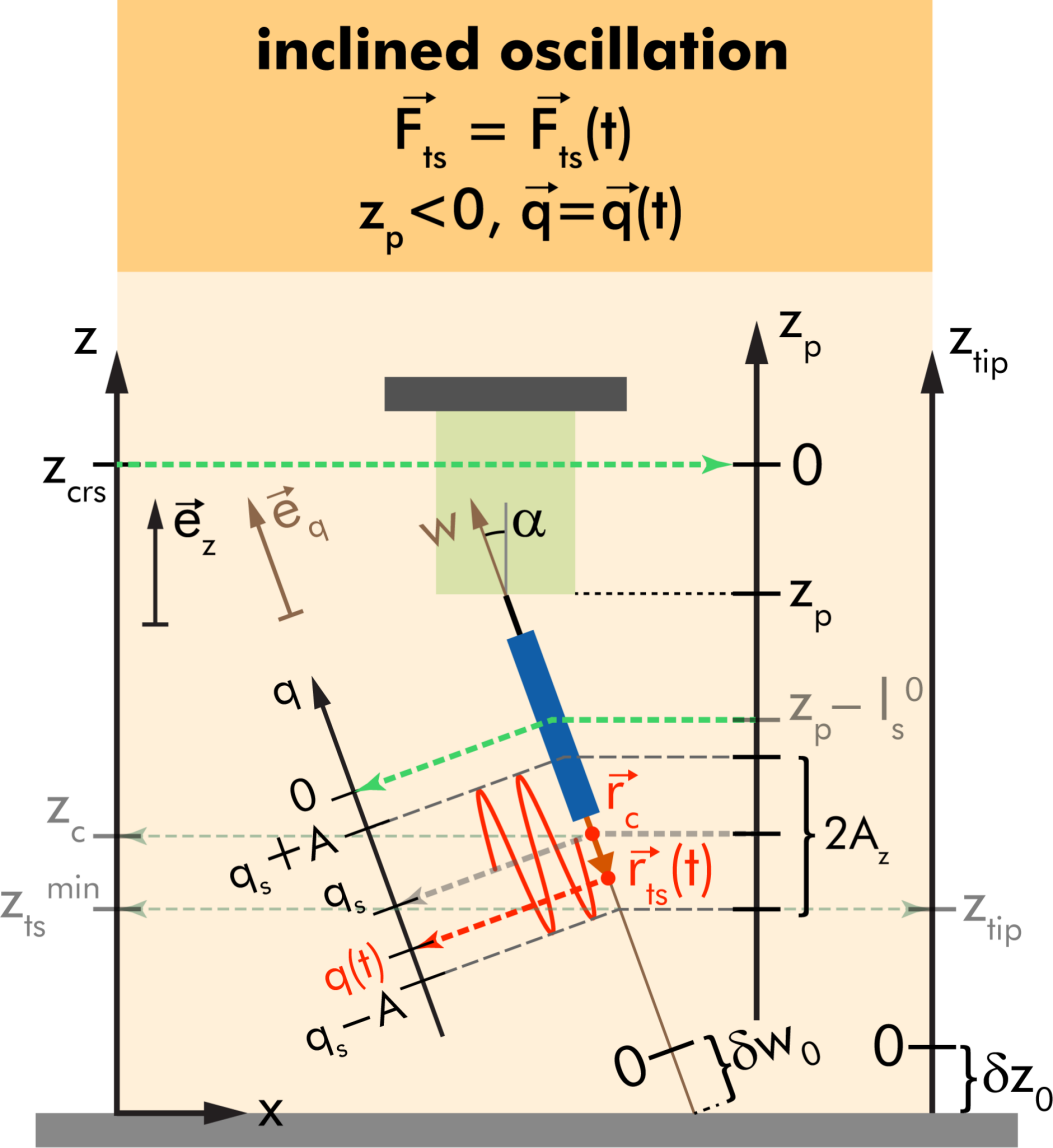
Coordinate system for considering an inclined oscillation by introducing the vector 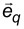 and the axis *w*.

A tip sampling path inclined relative to the *z*-axis implies that the direction of oscillation 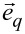 is tilted with respect to 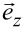 as illustrated in Figure 2. We introduce the inclined axis *w* parallel to the tip sampling path with 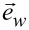 pointing in the direction of 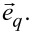 Assuming an inclination angle of α (with 0 ≤ α ≤ π/2) between 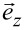 and 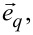 any position on the *w*-axis can be expressed by the respective position on the *z*_tip_-axis by a simple geometrical transformation. This implies that any sensor movement along *z*_p_ is not in line with the tip sampling path. Therefore, one has to take into account that the inclined oscillatory motion of the sensor can invoke significant lateral movement of the tip when describing the Δ*f* signal formation and force deconvolution. If the force field 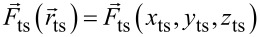 above the surface is homogeneous and isotropic with respect to the lateral coordinates *x*_ts_ and *y*_ts_, the inclined axis of sensor oscillation can be taken into account by using transformed position variables *z*_p_ → *z*_p_ cosα or *z*_tip_ → *z*_tip_ cosα.

If no such homogeneity is present, however, the *w*-axis has to be taken explicitly into account. The definition of a zero position of this *w*-axis goes along the same lines as the definition of zero δ*z*_0_ for the *z*_tip_-axis by introducing 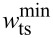 and the uncertainty δ*w*_0_.

For the further discussion, we define the vectorial sensor displacement 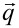 as


[1]

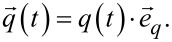



Within the harmonic approximation [3], *q*(*t*) is given as


[2]

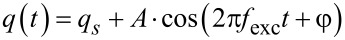



with the static deflection *q_s_*, the oscillation amplitude *A*, the excitation frequency *f*_exc_, and the phase φ [3]. In its vectorial form, the momentary position of the tip 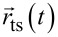 is given as


[3]

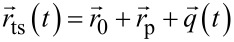




[4]

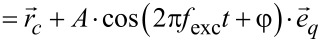



with the centre position 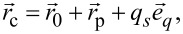 start position 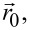 and piezo position 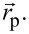 These quantities generalise the previously introduced *z* coordinates *z*_c_, *z*_0_, and *z*_p_, respectively. We further introduce the reduced amplitude *A*_z_ as the projection of *A* on the surface normal [2]


[5]

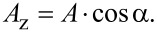



### Equation of motion for the inclined sampling path

Next, we derive the three AFM equations [3] linking the AFM physical parameters with the experimental observables and excitation parameters for a straight tip sampling path with arbitrary oscillation direction. The starting point is the differential equation describing the displacement *q*(*t*) in presence of the tip–sample force field 
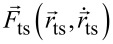
 and excitation force 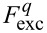 as follows


[6]

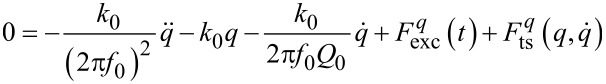



with the sensor parameters fundamental eigenfrequency *f*_0_, modal sensor stiffness *k*_0_ [18], and modal sensor quality factor *Q*_0_. This equation of motion is a one-dimensional differential equation depending on the tip–sample force component 
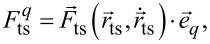
 following the description in [3,15–16]. The vectorial tip–sample force can generally be expressed by the sum of an even, 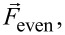 and an odd, 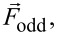 component


[7]

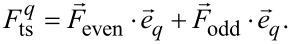



The deflection *q* is periodic with *T*_exc_ = 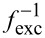 and the tip–sample force component 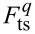 can, therefore, be expressed by the Fourier sum


[8]

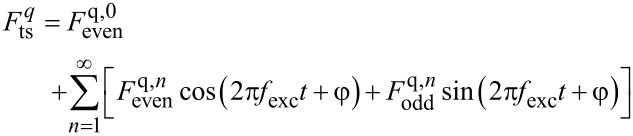



with the coefficient for *n* = 0


[9]

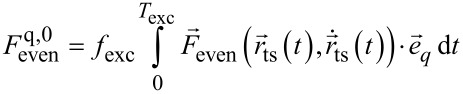



and the coefficients for *n* ≥ 1


[10]

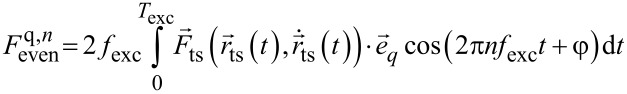




[11]

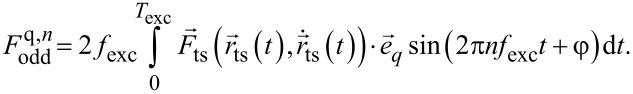



With the time average defined by [3]


[12]

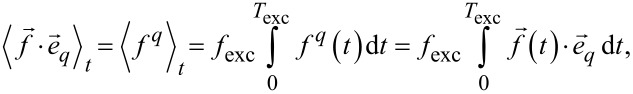



for an arbitrary function 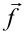 with projection 
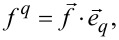
 the Fourier coefficients for *n* ≥ 1 can be expressed in terms of time averages


[13]

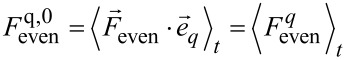




[14]

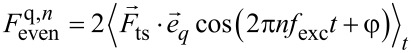




[15]

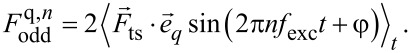



### AFM equations for the inclined sampling path

The three AFM equations follow from evaluating the Fourier coefficients 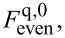 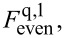 and 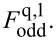 The first step is to calculate the time-averaged form of the three equations (see Appendix section for the derivations)


[16]

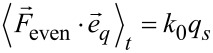




[17]

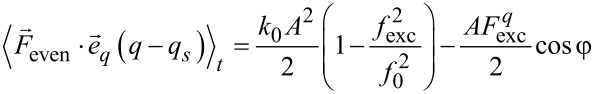




[18]

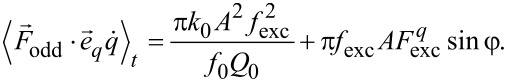



In a next step, the time averages are transformed to spatial averages similar to the formerly introduced cup and cap average functionals [3].

The harmonic approximation constrains the tip movement within the 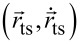 phase space to a closed trajectory. Consequently, the parametrisation with a spatial coordinate along this sampling path requires a parametrisation of the velocity by this coordinate as well. To reflect this dependency, we introduce the even force 
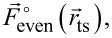
 formally defined by 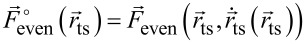 as the force along the tip sampling path. Then, we further define the projection of an arbitrary function 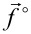 along the tip sampling path on the oscillation direction 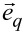 as 
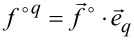
 and perform the integration along the sampling path symmetrically to the centre position 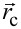 of this projected quantity 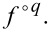 The cup and cap averaging functionals are then written as


[19]

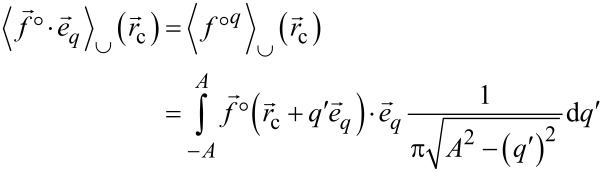




[20]

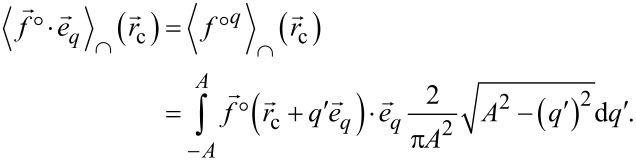



These averages have now the structure of line integrals along the tip sampling path parallel to 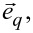 spanning the range −*A* to *A* as parameterised by *q*′.

We furthermore define the tip–sample force gradient along the oscillation path, 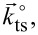 by the derivation of the force along the oscillation direction, namely


[21]

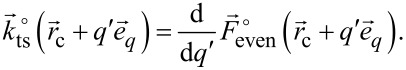



The three AFM equations follow now from Equation 16, Equation 17, and Equation 18 as


[22]

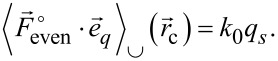




[23]

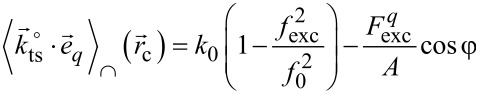




[24]

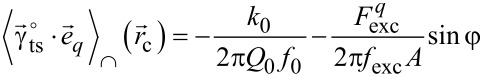



whereby the vectorial damping coefficient 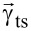 and the damping coefficient 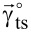 along the oscillation path have been introduced to write the odd force as


[25]

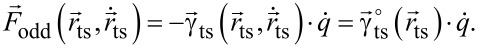



### Force response for the inclined sampling path

By reinterpreting the cup and cap averaging functionals as line integrals along the inclined tip sampling path, three AFM equations were found that represent the general case for a probe oscillating in an arbitrary direction. A probe orientation different from the surface normal and its oscillation in the vector force field above the surface has important consequences on the measured force response and appropriate data analysis procedures.

We demonstrate these consequences by simulating the frequency shift Δ*f* = *f*_exc_ − *f*_0_ in the frequency-modulated AFM mode for different cases using a Morse potential


[26]

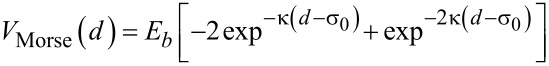



as a model that describes the interaction between two atoms at a distance *d* by the parameters *E_b_* = 0.371 aJ, σ_0_ = 0.235 nm, and κ = 4.25 nm^−1^ (adapted from [20]). We use this model for the pairwise interaction between a tip with a heterogeneous surface section. The surface section is built by arranging *N_a_* = 5 atoms at *z*_ts_ = 0 nm along the *x*-axis (with unit vector 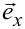) at an atom–atom distance of *d_a_* = 0.5 nm. To model a second atomic species for the heterogeneous surface section, *E_b_* of the central atom is scaled by a factor of four. A sixth probe atom at position 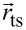 representing the tip is moved within the force field 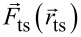 calculated from


[27]

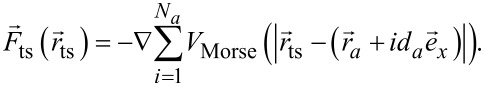



**Figure 3:**
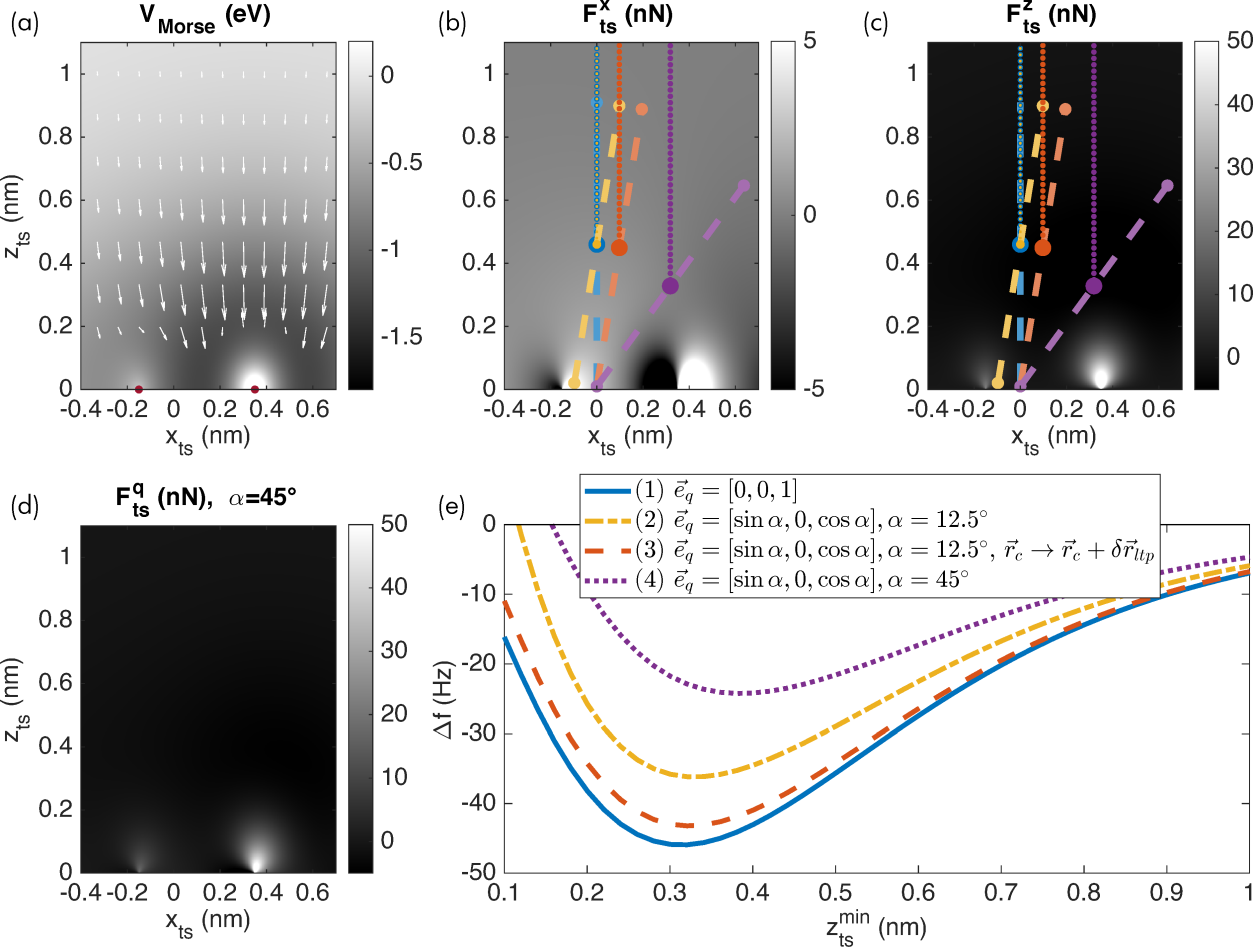
(a) Potential, (b) lateral force component, and (c) vertical forces for a pairwise Morse interaction summed over five surface atoms (positions of two atoms indicated by red points in (a), further atoms are located outside of the shown region to model a surface section). The sampling paths along the oscillation (dashed lines) as well as data recording paths (dotted lines) are included for four cases in (b,c). (d) Projection of the interaction force on the 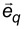-axis. (e) Δ*f*(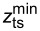) curves calculated for four different inclination angles and starting points.

Vector 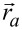 defines the origin of the surface section. In the following, the central atom is placed at 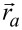 = (*x*_ts_, *y*_ts_, *z*_ts_) = (0.35, 0, 0) nm. The potential *V*_Morse_ and the force components 
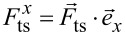
 as well as 
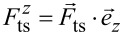
 are shown in Figure 3a, b, and c, respectively. A vectorial representation of the force field in the *x*_ts_–*z*_ts_ plane is included in Figure 3a.

To illustrate the effects resulting from an inclined tip oscillation, four cases are discussed. Common to all cases is that the data recording path, described by the oscillation centre positions 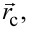 remains oriented parallel to the 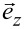-axis, that is, perpendicular to the surface as indicated by the dotted lines in Figure 3b and Figure 3c. This represents the common experimental protocol. In turn, the sampling path describing the tip oscillation is inclined by different angles α within the *x*_ts_–*z*_ts_ plane with the normalised inclined oscillation vector 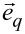 = [sinα, 0, cosα]. The tip trajectories during single oscillation cycles at one fixed 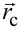 are indicated for each case by dashed lines in Figure 3b and Figure 3c.

The force component 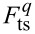 along the tip path is a scalar quantity and shown for α = 45° in Figure 3d. Compared to the vertical component 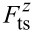 (see Figure 3c), the shape at the atom positions is asymmetric and the absolute contrast is diminished as a result of projecting the vectorial force 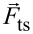 onto 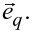


The force gradient 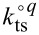 along the tip path and projected to 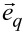 is calculated by numerical differentiation along 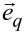 of the 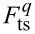 force field. The result is used to calculate frequency shift Δ*f* data from Equation 23 for φ = −π/2. As an example, we use parameters for a sensor often used in low-temperature environments (tuning fork sensor [21] with *f*_0_ = 30 kHz, *k*_0_ = 1800 N/m, and *A* = 0.45 nm). However, similar effects can be present when using parameters for other sensors as well. Frequency shift Δ*f* data are calculated with the piezo axis located at *x*_ts_ = *y*_ts_ = 0 and moving the tip along *z_p_* for data recording, while data are plotted as a function of 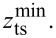


The solid blue curve in Figure 3e represents case (1) of a perpendicular oscillation with 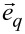 = [0,0,1]. When positioning the tip along the 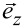-axis for data acquisition, this case allows for a reliable determination of the interaction force 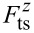 by applying known inversion strategies [2,4–5].

Next, the tip inclination is set to α = 12.5° within the *x*_ts_–*z*_ts_ plane as case (2) shown in yellow in Figure 3b and Figure 3c. The corresponding Δ*f*^(2)^(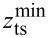) curve (dash-dotted yellow in Figure 3e) is different from the blue Δ*f*^(1)^(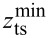) curve. This is expected as the lower turning point moved sideways and the cap averaging is performed along a different path than in case (1). Note that in contrast to case (1), the tip sampling path has no overlapping segments when moving the tip along *z*_p_. In case (3), the lateral movement of the lower turning point is compensated by subtracting the vector 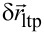 = [Δ*x*, 0, Δ*z*] with Δ*x* = −*A*sinα and Δ*z* = *A*(1 − cosα) from 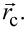 The resulting Δ*f*^(3)^(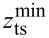) data included as a dashed red curve in Figure 3e deviates from all other curves.

When further increasing the inclination angle α as in case (4), the deviation becomes larger as presented by the violet dotted curve in Figure 3e for α = 45°. Last, we note that lateral components are virtually absent for large tip–sample distances in this model, leading to a convergence of the Δ*f*(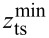) curves in the regime 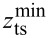 ≫ 1 nm.

### Force deconvolution for the inclined sampling path

**Figure 4:**
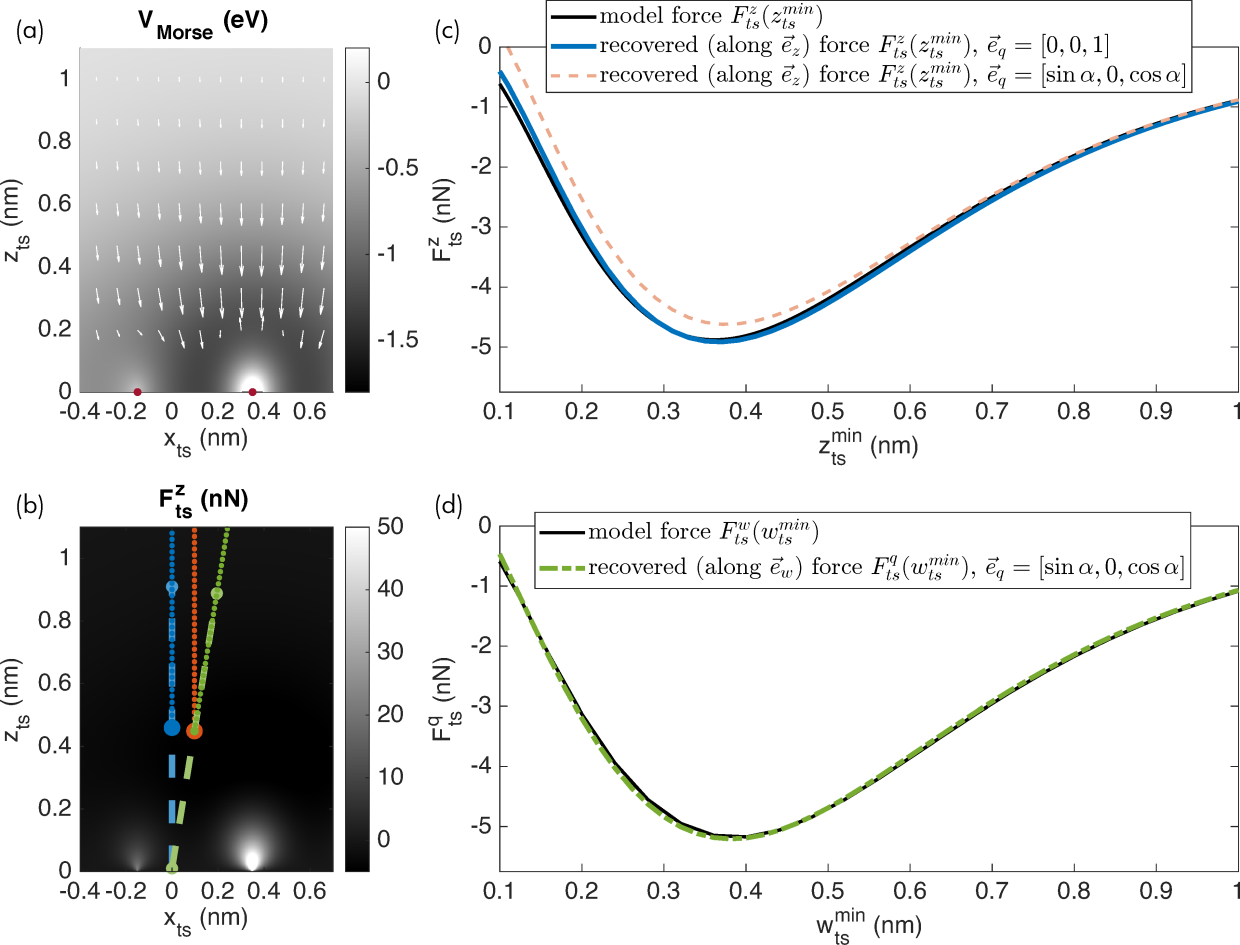
(a) Heterogeneous surface potential with the tip–sample force vector field indicated by arrows (same as Figure 3a). (b) Vertical force component with the tip sampling path (dashed lines) and data recording path (dotted lines). (c) Tip–sample forces plotted with respect to the vertical coordinate 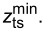 (d) Tip–sample forces plotted with respect to the parameter 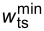 along the inclined oscillation direction. Remaining small deviations between the (c) black and blue curves and (d) black and green curves are explained by the approximations present in the Sader–Jarvis algorithm [20].

The difference in the orientation of 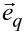 and 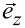 violates a fundamental assumption of the commonly used inversion algorithms [4–5]: The tip sampling path segments are not overlapping when moving the tip along the data recording path for an inclined oscillation. The resulting error in the force recovery is shown in Figure 4c, where the red dashed curve presents the recovered force for the case of an oscillation inclined by α = 12.5° and Δ*f* data recorded along 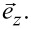 As is apparent, the force curve does not match the model reference curve, 
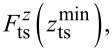
 included as the solid black line. In contrast, the force curve recovered for the vertical oscillation and vertical data recording (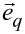 = [0,0,1], blue curve) matches the reference curve.

As a solution to this issue, we propose to orient the recording path for acquiring the AFM observables and parameters parallel to the tip sampling path 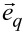 describing the tip oscillation. This modification leads to an overlap of the tip sampling path segments for nearby positions along the data recording path. Therefore, the deconvolution using the known algorithms can be performed in the usual manner. Naturally, the result will not represent the perpendicular force 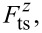 but rather describes the force component 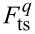 along the *w*-axis, parameterised by the scalar variable 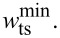 For a conservative force field, the vertical interaction force could in principle be calculated from this result. Additionally, if the full force field is of interest, this can be extracted by systematic measurements of many Δ*f* curves using the appropriate experimental procedures [22].

Simulation results for moving the tip along the inclined path during data acquisition and extracting the force along this path are presented in Figure 4d by the green curve. The force along this data recording path is correctly recovered as shown in Figure 4d where the green dash-dotted curve closely matches the model curve (in solid black) extracted along this path. Note that the force along an inclined *w*-axis is different from the vertical interaction force along 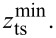


## Conclusion

Several conclusions can be drawn from extending the mathematical description of dynamic force microscopy by arbitrary tip sampling and data recording paths. For a typical inclination of α = 12.5°, the minimum force was calculated to differ by more than 5% when compared to a result not taking the inclination into account. The magnitude of this difference depends on the model parameter choice and geometry: The difference can be amplified or reduced depending on the oscillation amplitude, on the interaction potential strength and decay, as well as on the atomic geometry. For example, edges of finite atomic slabs or larger atomic clusters generate significant effects. In practice, a model calculation is required to determine the uncertainty in the measured force due to the inclined tip oscillation.

Precise forces are measured if the data recording path, here introduced as the axis *w*, is aligned parallel to the tip sampling path, here described as the vector 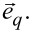 The resulting measured force represents the component 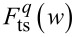 of the tip–sample force along this direction. Despite the formal and quantitative difference from the commonly considered vertical component 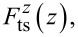 the component along *w* delivers identical physical insights into the tip–sample interaction.

## Appendix: Mathematical Derivations

### AFM Equation 1

The first AFM equation follows from evaluating the Fourier coefficient 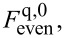 defined by


[28]

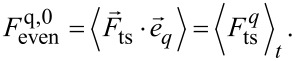



The tip–sample force can furthermore be written as a sum of an even and an odd force


[29]

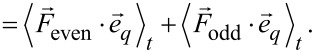



By definition of an odd force, the time average 
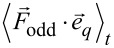
 evaluates to zero. We compare this equation by introducing the equation of motion (Equation 6) for 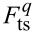 and using the fact that the time average is a linear functional


[30]

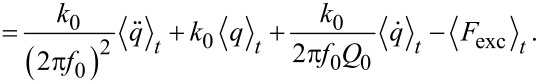



With the harmonic approximation (Equation 2) it can directly be shown that 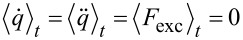 and *q_s_* = ⟨*q*⟩*_t_*. The first AFM equation directly follows as


[31]

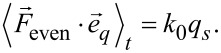



### AFM Equation 2

The Fourier coefficient 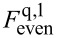 is defined as


[32]

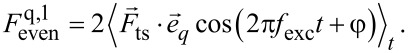



Within the harmonic approximation (Equation 2), this term can be written as


[33]

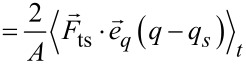



and 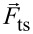 be expressed by even and odd forces


[34]

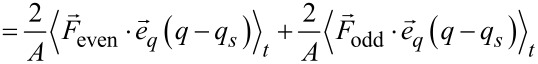



whereby the average 
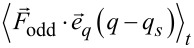
 evaluates to zero. Using the equation of motion (Equation 6), the Fourier coefficient can be written as


[35]

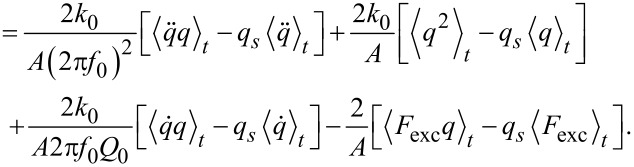



In full analogy to [3], this equation evaluates to


[36]

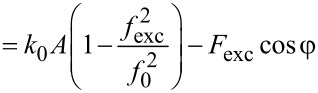



whereby the identities 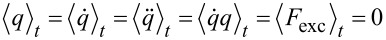 are used.

### AFM Equation 3

The Fourier coefficient 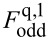 is defined as


[37]

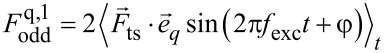



which can be written as


[38]

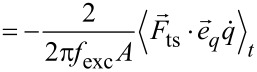



by using the harmonic approximation, Equation 2. The force is again expressed as a sum of even and odd contributions


[39]

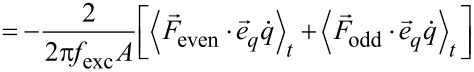



whereby 
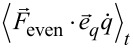
 evaluates to zero. Using the equation of motion, Equation 6, this is equal to


[40]

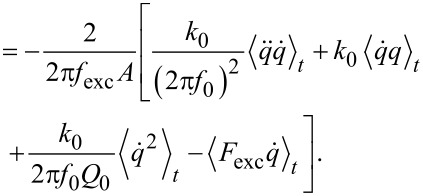



With the identities 
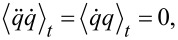
 this term evaluates to


[41]